# The Buffalo Sanatory Club

**Published:** 1897-03

**Authors:** 


A Monthly Review of Medicine and Surgery.

*EDITORS:*

THOMAS LOTHROP, M. D. - - WM. WARREN POTTER, M. D.

All communications, whether of a literary or business nature, should be addressed to the managing editor: 284 FRANKLIN STREET, BUFFALO, N. Y.

VOL. XXXVI. MARCH, 1897. No. 8.

### THE BUFFALO SANATORY CLUB.

A NEW SOCIETY for the acquisition and dissemination of knowledge relating to the preservation of health was recently organised in Buffalo, and to the best of our information and belief is the first of its kind in the world.

To many of our readers this statement will seem less than luminous—but let us see. This new club, or possibly this newer club, was formed at the instance of medical men, and by the suggestion of medical men—physicians of all so-called schools, or of no school at all, are invited to join its ranks and on a common level to labor for the accomplishment of its purposes. More than this, any person so interested may join this club, and we understand that special effort will be made to induce editors, reporters, microscopists, architects, builders, plumbers, heaters, men and women to join, and to study and work, each in his or her particular way, to gain or spread that greatly-to-be-desired knowledge, the best means for the preservation of health.

The JOURNAL, at present, is simply inclined to claim for the Sanatory Club of Buffalo the distinction of being the first of its kind in the world, and we wish its promoters all possible success.

The Sanatory Club proposes to hold six meetings during the year and at each meeting to discuss some topic in sanitary science; the first of such meetings will be in April, and the topic will be The water supply of Buffalo.

It is proposed that representatives of the press be asked to attend at the deliberations of the club, and we doubt not, by a wise selection of subjects and earnest practical and scientific discussion of the same, that matter will be produced worth reporting and worth reading.

We have for years held the conviction that sanatory knowledge in the study, the laboratory, the sanctum, was too far in advance of the information of the common people ; we, therefore, heartily welcome this new departure, believing that societies like this on popular democratic lines can perform an important part in the dissemination of the facts of sanitation.

